# Theoretical Description of R–X⋯NH_3_ Halogen Bond Complexes: Effect of the R Group on the Complex Stability and Sigma-Hole Electron Depletion

**DOI:** 10.3390/molecules25030530

**Published:** 2020-01-25

**Authors:** Juan Zurita, Vladimir Rodriguez, Cesar Zambrano, Jose Ramón Mora, Luis Rincón, F. Javier Torres

**Affiliations:** 1 Instituto de Simulación Computacional (ISC-USFQ), Universidad San Francisco de Quito, Diego de Robles y Vía Interoceánica, Quito 17-1200-841, Ecuador; jzurital@estud.usfq.edu.ec (J.Z.); vrodriguez@usfq.edu.ec (V.R.); czambrano@usfq.edu.ec (C.Z.); jrmora@usfq.edu.ec (J.R.M.); lrincon@usfq.edu.ec (L.R.); 2 Departamento de Ingeniería Química, Grupo de Química Computacional y Teórica (QCT-USFQ), Universidad San Francisco de Quito, Diego de Robles y Vía Interoceánica, Quito 17-1200-841, Ecuador; 3 Departamento de Matemáticas, Universidad San Francisco de Quito, Diego de Robles y Vía Interoceánica, Quito 17-1200-841, Ecuador

**Keywords:** sigma-hole, halogen bond, fermi-hole, conditional pair density, Kullback–Leibler divergence

## Abstract

In the present work, a number of R–X⋯NH_3_ (X = Cl, Br, and I) halogen bonded systems were theoretical studied by means of DFT calculations performed at the ωB97XD/6-31+G(d,p) level of theory in order to get insights on the effect of the electron-donating or electron-withdrawing character of the different R substituent groups (R = halogen, methyl, partially fluorinated methyl, perfluoro-methyl, ethyl, vinyl, and acetyl) on the stability of the halogen bond. The results indicate that the relative stability of the halogen bond follows the Cl < Br < I trend considering the same R substituent whereas the more electron-withdrawing character of the R substituent the more stable the halogen bond. Refinement of the latter results, performed at the MP2/6-31+G(d,p) level showed that the DFT and the MP2 binding energies correlate remarkably well, suggesting that the Grimme’s type dispersion-corrected functional produces reasonable structural and energetic features of halogen bond systems. DFT results were also observed to agree with more refined calculations performed at the CCSD(T) level. In a further stage, a more thorough analysis of the R–Br⋯NH_3_ complexes was performed by means of a novel electron localization/delocalization tool, defined in terms of an Information Theory, IT, based quantity obtained from the conditional pair density. For the latter, our in-house developed C++/CUDA program, called KLD (acronym of Kullback–Leibler divergence), was employed. KLD results mapped onto the one-electron density plotted at a 0.04 a.u. isovalue, showed that (i) as expected, the localized electron depletion of the Br sigma-hole is largely affected by the electron-withdrawing character of the R substituent group and (ii) the R–X bond is significantly polarized due to the presence of the NH_3_ molecule in the complexes. The afore-mentioned constitutes a clear indication of the dominant character of electrostatics on the stabilization of halogen bonds in agreement with a number of studies reported in the main literature. Finally, the cooperative effects on the [Br—CN]_n_ system (*n* = 1–8) was evaluated at the MP2/6-31+G(d,p) level, where it was observed that an increase of about ~14.2% on the complex stability is obtained when going from *n* = 2 to *n* = 8. The latter results were corroborated by the analysis of the changes on the Fermi-hole localization pattern on the halogen bond zones, which suggests an also important contribution of the electron correlation in the stabilization of these systems.

## 1. Introduction

**Scheme 1:**
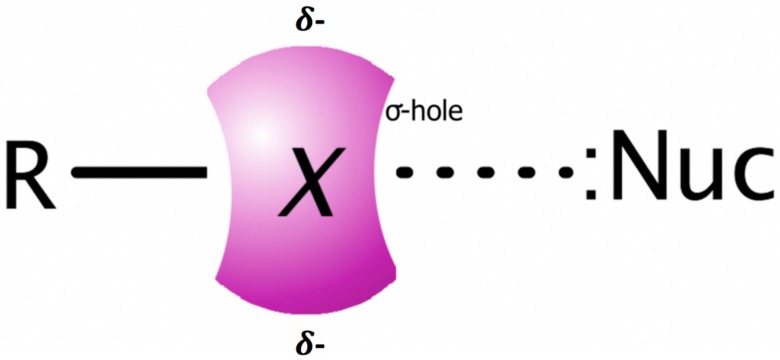
Schematic representation of the interaction of a nucleophile and a sigma-hole. The δ-purple zone represents the accumulation of electron density at zones perpendicular to the sigma-axis.

Halogen bond refers to the interaction between a nucleophile, Nu, and the heavy halogen atom belonging to a R–X bond (X = Cl, Br or I, and R = an organic moiety) [1,2,3,4,5,6,7]. This is a non-covalent interaction, denoted as R–X⋯Nu (see Scheme 1), where the nucleophile is usually aligned with the R–X bond axis, resulting in an angle ∡RXNu close to 180°. Experimental data retrieved from the analysis of a large number of crystalline structures published in the Cambridge Structure Database [8,9,10], have corroborated that halogen bonds are formed preferentially along the orientation of the sigma-axis [11,12]. Moreover, it has been experimentally determined that halogen bond exhibit red-shifts in the R–X stretching frequency upon complexation [13,14,15]; however, blue-shifts have also been observed in some cases. The latter is of significant interest because it indicates that this interaction cannot be described solely in terms of an electrostatic model. However, it has to be considered that the IR detection of halogen bonds is by no means a trivial task due to the strong coupling of the R–X stretching vibration with other normal modes; therefore, in most of the cases, no “pure” R–X vibrational modes exist, a fact that could affect the experimental measurements.

The halogen bond has attracted great attention in recent years since its presence is ubiquitous in the assembly of many functional materials [16]. Furthermore, it has been determined as one of the fundamental ingredients in various fields, such as crystal engineering, nanotechnology, and drug design [17,18,19,20,21,22,23]. From a theoretical point of view, the existence of the halogen bond seems surprising because both the nucleophile and the halogen atom involved in the interaction have negative charges; thus, according to classical electrostatics, these species should repel rather than form a stable complex [24]. A very elegant explanation for the occurrence of halogen bonds was attained by the introduction of the sigma-hole concept proposed by Politzer et al. [2,24,25,26,27,28,29,30,31,32,33,34], who used this term to refer to the zones of positive values of the electrostatic potential which are observed in the axial direction of an R–X bond (see Scheme 1). Within this concept, it is argued that the positive values of the electrostatic potential are the result of a significant depletion in the electron density localized in this sigma-axis zone. The observation of the sigma-hole confers to halogen bonds a dominant electrostatic character, similar to the one present in classical hydrogen bonds. Nonetheless, the first theoretical explanation for halogen bonds proposed by Mulliken in the 1950s [35] indicated that the halogen bonding stability is due to the charge transfer from the nucleophile lone pair to the R–X σ* orbital. Although this charge transfer mechanism contrasts with the sigma-hole electrostatic explanation, the former was confirmed in numerous theoretical studies of halogen bonded systems [8,10,36,37,38,39]. Of course, as observed in the hydrogen bond case, it seems feasible that the halogen bond could be explained as the interplay of both “charge transfer” and “sigma-hole electrostatic” interactions, these two mechanisms being ideal only in extreme situations. Indeed, recent studies have also proposed a significant role of dispersion in the stability of halogen bonds [38]. In order to explore the electronic behavior underlying in halogen bond formation, quantum mechanical methods have been often used; however, these have yielded a variety of results, which are, in some cases, contradictory. The latter is because, to date, there are no reported methods able to cope with all the important components (charge transfer, electrostatic and dispersion) [40] involved in the halogen bond mechanism of formation. One of the most accurate methods that has been successfully employed is the non-iterative coupled clusters with triple excitations CCSD(T) [21,36,41], whose results were used to estimate binding energies in relatively large molecular non-covalent complexes, which are in good agreement with results employing fully iterative benchmark methods [39] in more modest models. Other methods based on perturbation theory, such as MP2 and MP3 [42], also produced accurate enough results on non-covalent bound systems in comparison to fully iterative methods. Moreover, these two methods have been shown to reproduce experimental observations on complexes between halogenated molecules and many bases considering parameters such as equilibrium bond distances, dipole moments, polarizability, harmonic vibrational frequencies, inter- and intramolecular distances, for which experimental measurements are available. For the sake of comparison between theoretical and experimental results, Kozuch and co-workers [43] have proposed benchmark sets of molecules; namely: XB18 and XB51, that could be used to evaluate the accuracy of computational methods. By employing these benchmark sets, they have suggested that functionals with high exact-exchange and long-range corrections, especially M06-2X and ωB97XD, are the most suitable ones within the DFT context.

In order to get additional insights on the stability of the halogen bond complexes, different R–X⋯NH_3_ (X = Cl, Br, and I) systems are described in the present study by means of DFT and MP2 calculations. As a first stage, the effect of the electron-donating or electron-withdrawing character of the different R substituent groups (R = halogen, methyl, partially-fluorinated methyl, perfluoro-methyl, ethyl, vinyl, and acetyl) on the stability of the halogen bond is attained (see below). Then, a novel information-theory, IT, derived method of electron localization/delocalization [44,45,46,47] is employed in order to analyze the information content of the conditional pair density of a representative group of R–X⋯NH_3_ complexes. Through the latter analysis, a graphical representation as well an estimated quantitative measure of the electron depletion directly associated with the sigma-hole present in the different halogen bond systems are obtained, allowing us to retrieve conclusions on the various mechanisms controlling the formation and stability of these systems.

## 2. Results and Discussion

### 2.1. Relative Stability of the R–X⋯NH_3_ Systems

BSSE-corrected binding energies computed at the DFT are reported in Table 1 where it is observed that all the complexes under investigation are stable with BE^DFT^ values ranging from 1.0 to 16.4 kcal/mol, being the CH_3_Cl⋯NH_3_ system the only exception with a modest negative value of −0.1 kcal/mol. Beyond this general picture, two clear tendencies are observed in Table 1: (i) for a given R substituent group, the relative stability of the complexes follows the Cl < Br < I trend indicating that in agreement with previous reports [1], the less electronegative the halogen bond-donor, the larger the stability of the halogen bond complex; and (ii) for any halide atom acting as halogen bond donor, the relative stability with respect to the R substituent groups follows the –X > –CF_3_ > –CHF_2_ > –CH_2_F > –CH_3_ trend, suggesting that, apart from the –X substituent, the higher the electron-withdrawing character of R the larger the stability of the complex. Regarding the effect of the same halide atoms acting as R substituent groups, it is clear that their effect on the stability does not follow the aforementioned rule and the electronegativity of the halogen bond donor has a more significant role in the complex stability for the X_2_⋯NH_3_ cases. In agreement with the latter tendencies, the distance of the halogen bond D_X⋯N_ decreases accordingly with the relative stability, being the shortest D_X⋯N_ value found in the case of the X_2_⋯NH_3_ systems and the largest one in the CH_3_X⋯NH_3_ cases. Comparison with the BSSE-uncorrected BE^DFT^ results (numbers in parenthesis of Table 1) shows that the BSSE accounts for less than 1.0 kcal/mol for both the R-Cl⋯NH_3_ and R-I⋯NH_3_ cases; whereas the error values for the R-Br⋯NH_3_ systems are larger, being within the 1.0–3.7 kcal/mol range. This indicates that the 6-31+G(d,p) basis set employed for Br can be considered fairly incomplete in comparison to the Cl atom. Moreover, the incompleteness of the basis set in the case of the I atom is somehow compensated through its description in terms of the LANL2DZ ECP.

**Figure 1:**
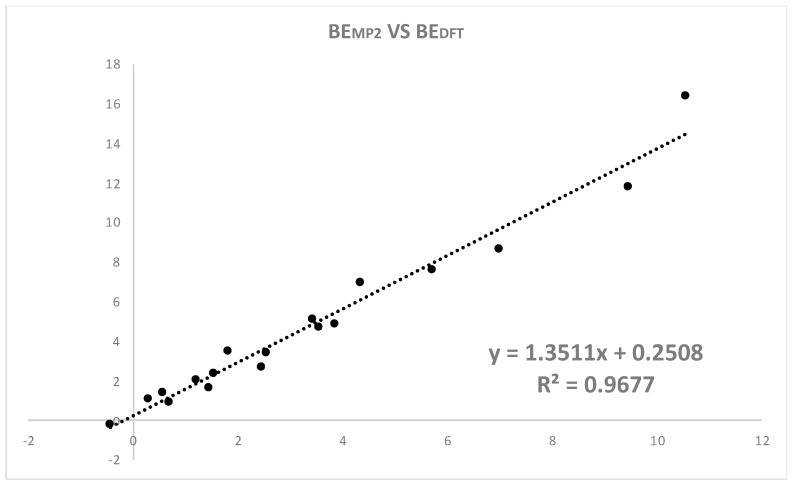
Correlation plot of BE^MP2^ against BE^DFT^ computed for the different R-X⋯NH_3_ complexes. Computed values for the R = ethyl, vinyl, and acetyl considered for the Br-bond systems are also included in the Figure. Energy values reported in kcal/mol.

Inspection of the BSSE-corrected binding energies obtained at the MP2 level (Table 1) indicates that this correlated-level of calculation provides, in general, less stable complexes of which the R-I⋯NH_3_ systems are the ones presenting the largest ΔBE = BE^MP2^−BE^DFT^ differences, ranging from −1.7 to −5.9 kcal/mol. For the case of the R-Br⋯NH_3_ complexes, the ΔBE differences are more homogenously distributed, being within the −1.0 to −1.7 kcal/mol range, whereas ΔBE is almost constant to ~ −0.3 kcal/mol for the R-Cl⋯NH_3_ systems except for the Cl_2_⋯NH_3_ case for which a value of −2.6 kcal/mol is obtained. In spite of these differences, the BE^MP2^ data reported in Table 1 follows the same two trends observed for the BE^DFT^ values (see above). Indeed, a linear correlation analysis between of the BE^DFT^ and BE^MP2^ data results in a R^2^ value of 0.9677, confirming that the DFT description by means of the ωB97XD functional is quite capable to cope with the main energetic features of the various halogen bond systems explored in the present study (see Figure 1). Although the striking agreement of the two sets of date, two cases are be to further commented since they present the largest difference. A ΔBE of −2.6 and −5.8 kcal was obtained for the Cl_2_⋯NH_3_ and I_2_⋯NH_3_ systems, respectively, which could be attributed to the parametrization of the halogen atoms within the ωB97XD approach as well as the employment of a pseudo-potential for the I atom, both factors resulting in an overestimation of the BE at the DFT level. In agreement to the lower stability of the complexes computed at the MP2 level, the resulting D_X⋯N_ values are slightly larger by an average of ~0.05 Å with respect to their DFT counterparts. This modest change in the geometry suggests that any difference between the DFT and the MP2 results are due to the description provided by the two different levels and not due to geometric effects.

At this point, it is important to indicate that the use of a relatively small basis set (i.e., 6-31+G (d,p)) allow the further calculation of the $\chi_{XC}^{\sigma }$ and $\chi_{C}^{\sigma }$ quantities (see below) to be conducted at a moderate computational cost. In order to explore the effect of the increase of the basis set flexibility on the relative stability of the studied complexes, a further optimization of the systems was carried out at the MP2/6-311+G(d,p) level. As a result, a negligible change in the binding energies was observed for the complexes containing Cl and Br (i.e., between −0.2 and 0.2 kcal/mol), whereas an increase of about ~1.5 kcal/mol (i.e., between 1.3 and 1.7 kcal/mol) was determined for the complexes containing I. The latter can be attributed the use of a pseudo-potential for the I atom (i.e., LANL2DZ ECP). In spite of these changes in the MP2 computed binding energies, the same conclusions with respect to the relative stability of the different R–X⋯NH_3_ systems can be done (see above). In a further step, CCSD(T) /6-311+G(d,p)//MP2/6-31+G(d,p) calculations were carried out on the R–X⋯NH_3_ complexes. The resulting BSSE-free binding energies are summarized in Table 1, where it is observed that the tendencies of the complexes are kept regarding their relative stability, suggesting a good agreement between the MP2 and CCSD(T) description of the systems.

Changes in the NBO atomic charges of the halogen bond acceptor (N), halogen bond donor (X), and its first covalently bonded neighbor (XN) are also reported in Table 1. A negligible change in the N atom charge is observed at the DFT level for most systems as expected considering the weak X⋯N interaction. Only in the case of the X_2_⋯NH_3_ and R–I⋅⋅⋅NH_3_ systems a positive change for Δq_N_ is observed. MP2 results (data in parenthesis of Table 1) provide a more homogeneous picture in which the N charge remains almost unchanged upon the formation of the complexes. It must be indicated that the latter picture indicate that the X⋯N interaction could be essentially electrostatic [27,28] with a very small degree of charge transfer. On the other hand, Δ*q_X_* and Δ*qx_N_* values obtained at the MP2 level indicates that the halogen bond donor loses some charge density, which is in turn displaced to its first neighbor. The latter results in a polarization of the R–X bond induced by the presence of the NH_3_ molecule. In order to better understand this mechanism, KLD calculations on the R—Br⋯NH_3_ systems (i.e., complexes presenting neither the highest nor the lowest stability) are presented in the next section.

### 2.2. KLD Analysis of R-Br⋯NH_3_ Systems

**Figure 2:**
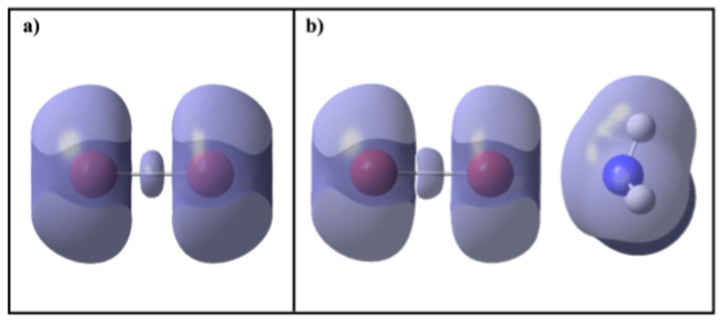
Graphical representation of the $\chi_{XC}^{\sigma }$ function computed at a 0.3 a.u. isovalue for (**a**) the Br_2_ isolated molecule and (**b**) and the Br_2_⋯NH_3_ complex.

**Figure 3:**
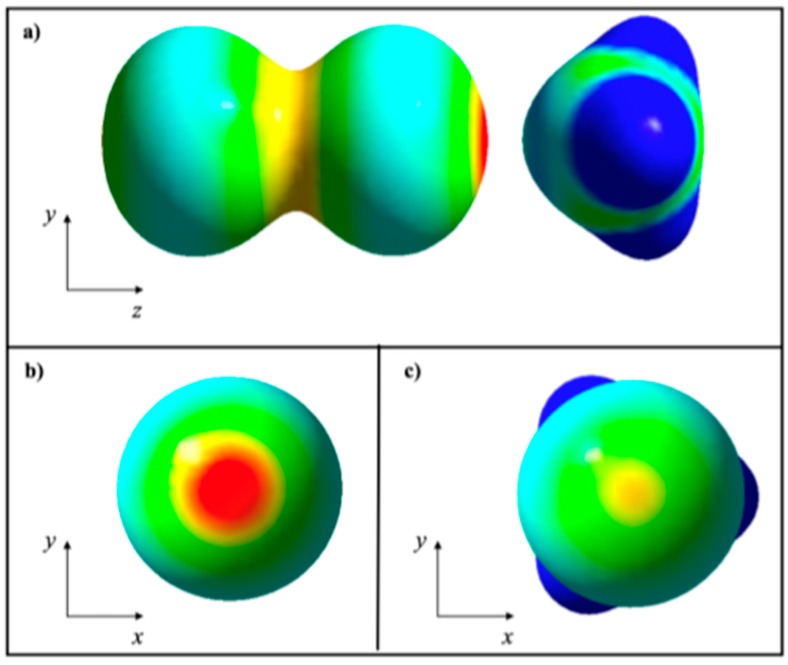
$\chi_{XC}^{\sigma }$ mapped onto the 0.04 a.u. isovalue of the electronic density for the the Br_2_⋯NH_3_ complex. The values range 0.2–0.5 bits goes from red to blue. Views perpendicular to the *yz* and *xy* planes are shown in the upper (**a**) and lower parts (**b**) and (**c**) of the figure, respectively. In Figure 3b, the NH_3_ molecule and its iso-value surface has been omitted for the sake of clarity.

In order to confirm the proposed polarization of the R–Br bond induced by the presence of the NH_3_ Lewis base, KLD calculations on the Br_2_ molecule and the Br_2_⋯NH_3_ complex (the system where the polarization is more evident) were performed. The tri-dimensional graphical representation of the $\chi_{XC}^{\sigma }$ function computed at a 0.3 a.u. isovalue for both systems is presented in Figure 2. In the Br_2_ isolated molecule, 9 basins are observed: 6 core basins that integrate to 56 electrons (3 basins for each Br atom that account for the core shells), 2 valence lone-pair ring basins that integrate to 11.44 electrons (one for each Br atom) and 1 bond basin located at the middle of the two atoms that integrated to 2.56 electrons. The two lone-pairs ring basins form a toroid-like shape whose void (at 0.3 a.u. isovalue) is oriented perpendicular to the bond axes, in agreement to the observations in the electrostatic potential which is customary interpreted in terms of the sigma-hole [2,24,25,26,27,28,29,30,31,32,33,34]. This means that the lone-pair electrons are localized perpendicular to the bond axis, creating an electron depletion in the sigma-axis. Regarding the Br_2_⋯NH_3_ complex, the computed $\chi_{XC}^{\sigma }$ function (Figure 2b) presents some differences in the basins when compared to the Br_2_ isolated molecule. In first place, it is observed that the bond electrons basin, that in this case integrates to 2.39 electrons, is located closer to the Br atom which is not directly involved in the halogen bond (i.e., acting as substituent group). This observation is consistent with the proposed polarization of the Br–Br bond induced by the presence of the NH_3_ Lewis base, and supports the well-established criterion on halogen bonds which states that this particular interaction is mainly governed by electrostatic effects rather than charge transfer or dispersion effects [27,28]. Analysis of the other two valence basins of the Br_2_ molecule of the complex shows that they are not identical in contrast to the case of the isolated molecule, being smaller the one associated to the Br atom that acts as halogen bond donor which loses some electron density. The Br ring basin of the halogen bond donor integrates to 5.56 electrons in the complex, whereas the ring basin of the opposite Br atom integrates to 5.77, this considerable difference constitutes a further evidence of the polarization of the bond. Moreover, this observation suggests that the sigma-hole present in the complex is being accurately accounted for by our KLD calculations. In a further step of this analysis, the $\chi_{XC}^{\sigma }$ function of the complex is mapped onto the one-electron density computed at a 0.04 a.u. isovalue. This particular isodensity, which is different to the isovalue customarily used for this analysis (i.e., 0.001 a.u. van der Waals limit [48]), was employed to obtain separated pictures of the density of each component of the complex (i.e., Br_2_ and NH_3_ molecules). Following the essence of the halogen bond analysis in terms of ESP maps [27], the $\chi_{XC}^{\sigma }$ result mapped onto the density is employed to furnish qualitative as well as quantitative evidence of the sigma-hole. The mapped function of the Br_2_⋯NH_3_ complex is shown in Figure 3, where zones of different electronic localization, lying within the 0.2–0.5 bits range, can be observed. As expected, the zone associated to the sigma-hole presents the lowest value of electron localization in agreement to the idea that a depletion of the electron density characterize this zone. On the other hand, the opposite side of the Br_2_ one-electron density has a larger $\chi_{XC}^{\sigma }$ value, denoting a higher electron localization at the border of the molecule, which is not involved in the halogen bond. Besides the Br_2_⋯NH_3_ complex, the same approach was employed for the analysis of the other R–Br⋯NH_3_ systems. For this stage of the study, the ethyl, vinyl, and acetyl substituents were included to evaluate the effect of having single, double, triple bonds in the R group on the stability of the halogen bond. As observed in Figure S1 (see Supplementary Information), a significant low $\chi_{XC}^{\sigma }$ value is obtained at the zone of Br sigma-hole, suggesting that for all cases an electronic depletion is located on the halogen bond donor. Moreover, the quantification of the $\chi_{XC}^{\sigma }$ value associated to the sigma-hole of the various R–Br⋯NH_3_ systems shows that the degree of electron delocalization depends on the electron-withdrawing character of the R group. The latter quantities along with the populations in the Br ring lone-pair basins are reported in Table 2, where it can be observed that the same trends, for both the minimum values of the $\chi_{XC}^{\sigma }$ and the population of the Br ring basin, hold. Clearly, there is a strong correlation between the degree of electron localization of the ring basin associated to the halogen donor, their electron population, and the stability of the halogen bond (BE^MP2^ values in Table 2). This result confirms the usefulness of the present electron localization measure to understand the trends in bonding (e.g., halogen bonding) that results from a combination of electrostatic and charge transfer mechanism.

### 2.3. KLD Analysis of [BrCN]_n_ Chains

**Figure 4:**
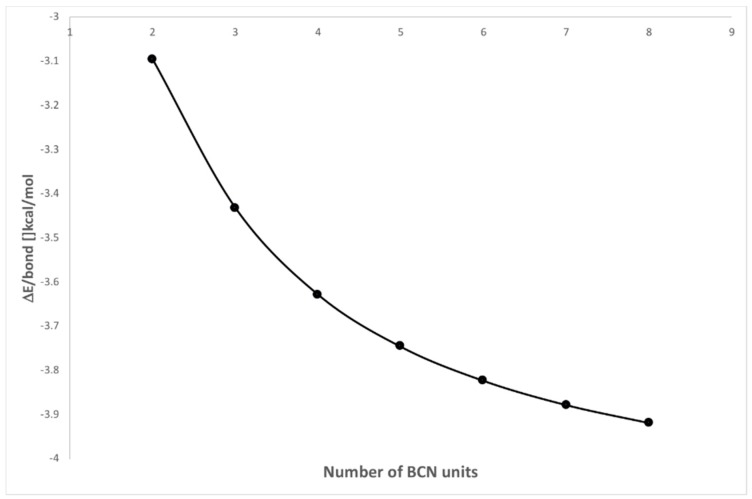
Change in the binding energy per bond computed for the different [Br—CN]_n_ systems.

**Figure 5:**
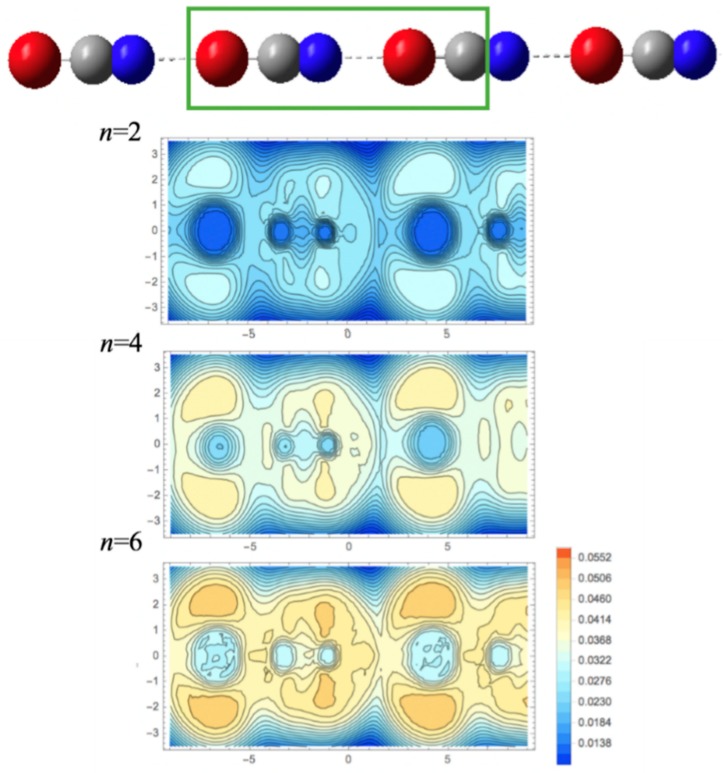
Contour plots of the $\chi_{C}^{\sigma }$ function computed for the [Br–CN]_n_ systems with *n* = 2, 4, and 6. The values range goes from 0.01 to 0.06 bits, corresponding to blue and red colors, respectively.

Although it has been well established that electrostatic effects are the dominant forces controlling the halogen bond complexes formation, charge transfer and dispersion effects have been also suggested to be important in the stabilization of these systems [38,49]. Following a previous work [50] and in order to analyze the effect of dispersion on the halogen bond complexes formation, chains composed of BrCN units, which can act as both halon-bond acceptor and donor, have been theoretical studied in the present work at the MP2/6-31+G(d,p) level. For complexes of [BrCN]_n_ with *n* = 2 to 8, the BSSE-corrected interaction energy per formed bond was computed in order to investigate the existence of possible cooperative effects in these chains. The resulting interaction energies per bond are presented in Figure 4, where it is observed that this quantity is not constant, and it decreases from a value of −3.1 to −3.91 kcal/mol computed for [BrCN]_2_ and [BrCN]_8_, respectively. At first sight, the latter change may seem to be negligible, yet it represents a stabilization of nearly 14.2%. Considering that, in a pure electrostatic regime (i.e., pair-wise additive potential), the interaction energy of per added unit in a complex system formed by several monomers tends to be constant, the latter result suggests a non-negligible contribution of dispersion effects in the stabilization of the halogen bonds present in the [BrCN]_n_ chains. This observation is in agreement with the results of a recent study [49], where dispersion was observed to play a significant role in halogen bond interactions. As proposed in a previous work [46], $\chi_{C}^{\sigma }$ is here employed as a tool to graphical represent the contribution of electron correlation in the BrCN⋯BrCN halogen bond. Before discussing the $\chi_{C}^{\sigma }$ results, it is important to mention that the linear arrangement [BrCN]_n_ chains allows the calculation of the $\chi_{C}^{\sigma }$ quantity in two-dimensional meshes adjoining the central BrCN⋯BrCN. $\chi_{C}^{\sigma }$ contour maps for the systems [BrCN]_n_ with *n* = 2, 4, and 6 are shown in Figure 5, where it is observed that the maximum values of the function located at the BrCN⋯BrCN halogen bond position increases with respect to the chain size, being this an indication of a stabilization effect of dispersion over the halogen bond formation.

## 3. Models and Methods

As mentioned above, the ability of different heavy halogen compounds R–X (with X = Cl, Br, and I; R= −X, −CH_3_, −CH_2_F, −CHF_2_, and −CF_3_) to form stable halogen bond complexes with NH_3_ was theoretical analyzed in the present work from the perspective of electron localization. It is important to remark that the NH_3_ molecule acting as halogen bond acceptor (i.e., nucleophile) was consider constant for all systems, because previous reports that combined data extracted from the Cambridge Structural Database and DFT-D3 theoretical results have suggested that halogen bonding interactions are energetically more favorable when amine electron donors are present [8]. As the first stage of the study, the R–X⋯NH_3_ complexes were fully optimized using the long-range corrected hybrid ωB97XD method, which includes a Grimme’s type correction that accounts for the dispersion energy [51], together with the 6-31+G(d,p) basis set. Only for the case of the systems containing the I atom, the LANL2DZ ECP basis set was employed. It is also worth of mentioning that the error due to the basis set superposition (BSSE) was corrected during the minimization of the energy with respect to the geometry by employing the Boys-Bernardi counterpoise method as implemented in the GAUSSIAN16 suit of programs [52]. Upon obtaining the DFT equilibrium structures, the results were reoptimized at the MP2 level by using the same basis sets described above, where the BSSE was also corrected during the optimization process. All the equilibrium geometries obtained at the two levels of theory were confirmed to be minima of the potential energy surface by means of a further BSSE-free vibrational analysis. CCSD(T)/6-311+G(d,p)//MP2/6-31+G(d,p) were also performed. The structural features as well as changes in the NBO atomic charges arising from the formation of the complexes were evaluated. Moreover, binding energies (i.e., negative of the stabilization energies or interaction energies, *IE*) were estimated for the three sets of results (i.e., DFT, MP2, and CCSD(T)) by means of the supermolecular approach:

(1) $$BE_{AB}=-IE_{AB}=-\left(E_{AB}-E_{A}-E_{B}\right)$$

where $E_{AB}$, $E_{A}$, and $E_{B}$ are the energies of the AB complex and its components A and B, respectively.

Among the computational approaches typically employed to reveal the sigma-hole contribution, one of the most applied is the electrostatic potential (ESP) mapped onto a certain isovalue (i.e., 0.001 a.u.) of the one-electron density [27]. The positive zones of ESP located on the terminal halogen atom along the R–X axis is the ubiquitous signature of the sigma-hole and its consequent ability to form stable complexes. In this work, a novel IT-derived quantity is proposed as a computational alternative to reveal the sigma-hole. For a thorough discussion of our method, the reader is referred to a recent review [47] and other recent reports [44,45,53]. Here, it is only reminded that our method requires the computation of the same-spin pair density, $\Gamma^{\sigma \sigma }\left(r_{1},r_{2}\right)$, which can be interpreted as the probability of finding an electron at $r_{1}$ with spin σ, and simultaneously a second electron at $r_{2}$ with the same spin. Within the mono-determinant approximation (expanded in terms of either Hartree-Fock or Khon-Sham orbitals), $\Gamma^{\sigma \sigma }\left(r_{1},r_{2}\right)$ can be computed as follows:

(2) $$\Gamma^{\sigma \sigma }\left(r_{1},r_{2}\right)=\rho^{\sigma }\left(r_{1}\right)\rho^{\sigma }\left(r_{2}\right)-{\left|\gamma^{\sigma \sigma }\left(r_{1},r_{2}\right)\right|}^{2}$$

The latter expression can be rewritten in the form:

(3) $$\Gamma^{\sigma \sigma }\left(r_{1},r_{2}\right)=\rho^{\sigma }\left(r_{1}\right)\gamma_{Cond}^{\sigma \sigma }(r_{2}|r_{1})$$

where $\rho^{\sigma }\left(r_{1}\right)$ is the one-electron density of σ-spin electrons and $\gamma_{Cond}^{\sigma \sigma }(r_{2}|r_{1})$ is the same-spin conditional pair density. In contrast to $\Gamma^{\sigma \sigma }\left(r_{1},r_{2}\right)$, $\gamma_{Cond}^{\sigma \sigma }(r_{2}|r_{1})$ can be interpreted as the probability of finding an electron at $r_{2}$ with spin σ, when it is known with certainty that a reference electron with σ spin is located at $r_{1}$ (notice the parametric dependence of the function on the position of the reference electron). The conditional pair density is customary written as follows:

(4) $$\gamma_{Cond}^{\sigma \sigma }\left(r_{2}|r_{1}\right)=\rho^{\sigma }\left(r_{2}\right)+\eta_{XC}^{\sigma \sigma }(r_{2}|r_{1})$$

where $\eta_{XC}^{\sigma \sigma }(r_{2}|r_{1})$ is defined as the same-spin correlation-exchange hole density, and it contains the information of how an electron at $r_{1}$ influences another electron at $r_{2}$ due to the Pauli principle. From Equation (4) it is clear that $\gamma_{Cond}^{\sigma \sigma }\left(r_{2}|r_{1}\right)$ is a distribution function that differs from the one-electron density $\rho^{\sigma }\left(r_{2}\right)$, only by the correlated-exchange hole term $\eta_{XC}^{\sigma \sigma }(r_{2}|r_{1})$. In order to have access to the information contained in the conditional pair density, IT-derived analysis tools can be employed. In these regards, the Kullback–Leibler divergence, $D_{KL}$, has been shown to present some advantages [45]. For a pair of distribution functions p(x) and q(x), $D_{KL}$ is defined as follows:

(5) $$D_{KL}(p||q)=\int p\left(x\right)log_{2}\frac{p\left(x\right)}{q\left(x\right)}dx$$

where the $log_{2}$ function is used to obtain $D_{KL}$ in bit units. $D_{KL}$ can be interpreted as the information lost when q(x) is used to approximate p(x). Furthermore, some important properties of $D_{KL}$ are: (i) it is always positive and (ii) it equals zero under the p(x)=q(x) in the whole space. If p(x) and q(x) in Equation (5) are respectively replaced by the following normalized distribution functions ρcondσσ=γCondσσ(r2|r1)Nσ−1 and σσ=ρσNσ, $D_{KL,XC}^{\sigma }$ can be defined as follows:

(6) $$D_{KL,XC}^{\sigma }\left(r_{1}\right)=\int dr_{2}\rho_{cond}^{\sigma \sigma }\left(r_{2}|r_{1}\right)log_{2}\frac{\rho_{cond}^{\sigma \sigma }\left(r_{2}|r_{1}\right)}{\sigma^{\sigma }\left(r_{2}\right)}$$

By taking into consideration the interpretation of the Kullback–Leibler divergence, $D_{KL,XC}^{\sigma }$ can be pictured as a measure of the information content in the exchange-correlation hole density $\eta_{XC}^{\sigma \sigma }(r_{2}|r_{1})$, and it can be applied to obtain the localization patterns of electrons in the molecular space. The latter is conceivable by considering that large values of $D_{KL,XC}^{\sigma }$ are associated with molecular zones in which the exchange-correlation hole is significant and vice versa. Finally, by including the scaling of the $D_{KL,XC}^{\sigma }$ with respect to $N^{\sigma }$, the following expression is introduced as a general descriptor of electron localization:

(7) $$\chi_{XC}^{\sigma }\left(r_{1}\right)=(N^{\sigma }-1)D_{KL,XC}^{\sigma }\left(r_{1}\right)f_{cut}\left(r_{1}\right)$$

where $f_{cut}\left(r_{1}\right)=\frac{1}{2}\left(1.0+ERF\left(0.5log_{10}\left(\frac{\rho \left(r_{1}\right)}{\rho_{cut}}\right)\right)\right)$ is introduced to truncate $D_{KL,XC}^{\sigma }$ at zones where the one-electron density is negligible and therefore, to obtain physical meaningful results [44].

Along the same vein, p(x) and q(x) in Equation (5) can be replaced by the normalized distributions $\rho_{cond,XC}^{\sigma \sigma }$ and $\rho_{cond,HF}^{\sigma \sigma }$, respectively, where the former one is obtained from a correlated level of theory, whereas the latter is computed from a Hartree-Fock wavefunction using the correlated geometry. It is clear that $\rho_{cond,XC}^{\sigma \sigma }$ presents certain difficulties in its evaluation, since correlated methods are typically based on a multiconfigurational wavefunction (i.e., more than one determinant). This limitation is circumvented in our method by considering the following approximation:

(8) $$\Gamma_{XC}^{\sigma \sigma }\left(r_{1},r_{2}\right)=\frac{1}{2}\left[\rho^{\sigma }\left(r_{1}\right)\rho^{\sigma }\left(r_{2}\right)-{\left|\gamma^{\sigma \sigma }\left(r_{1},r_{2}\right)\right|}^{2}+\Gamma_{C}^{\sigma \sigma }\left(r_{1},r_{2}\right)\right]$$

which can be envisaged as a generalized form of Equation (2), and it was previously proposed for the first time by Levi in 1987 [54]. The three contributions of Equation (8) can be expressed in terms of a natural orbital expansion as thoroughly discussed in [46]. Here, it is only recalled that, upon the calculation of $\Gamma_{xC}^{\sigma \sigma }\left(r_{1},r_{2}\right)$ from a correlated-method, $D_{KL,C}^{\sigma }$ can be readily computed through the following expression:

(9) $$D_{KL,C}^{\sigma }\left(r_{1}\right)=\int dr_{2}\rho_{cond,XC}^{\sigma \sigma }\left(r_{2}|r_{1}\right)log_{2}\frac{\rho_{cond,XC}^{\sigma \sigma }\left(r_{2}|r_{1}\right)}{\rho_{cond,HF}^{\sigma \sigma }\left(r_{2}|r_{1}\right)}$$

In contrast to $D_{KL,XC}^{\sigma }$, the effects of the exchange are absent in $D_{KL,C}^{\sigma }$; thus, this quantity reflects the electronic behavior due to correlation of same spin electrons, and it can be employed to compute the function $\chi_{C}^{\sigma }$ [46] through a similar expression as the one shown in Equation (7).

Both $\chi_{XC}^{\sigma }$ and $\chi_{C}^{\sigma }$ functions were computed from wavefunctions obtained with the GAUSSIAN16 suit of programs by employing our in-house developed C++/CUDA program, called KLD and compiled with NVIDIA CUDA release 10.1 running under Ubuntu 19.04. A standard NVIDIA GeForce GTX 1070 GPU was employed for the calculations, which were divided into four stream flows, each one of them, containing roughly the same number of points to be evaluated [55]. The KLD code is freely available upon request.

## 4. Conclusions

In the present study, various R–X⋯NH_3_ (X = Cl, Br, and I; R = halogen, methyl, partially-fluorinated methyl, perfluoro-methyl, ethyl, vinyl, and acetyl) halogen bond systems were theoretical studied by means of calculations performed at the ωB97XD level and the ab initio MP2 method (CCSD(T) results are also presented). In addition to this, a novel electron localization/delocalization IT-based method was employed to characterize the sigma-hole present in a representative group of these complexes (i.e., R–Br⋯NH_3_). DFT results showed that: (i) for a given R substituent group, the relative stability of the complexes follows the Cl < Br < I trend indicating that, in agreement with previously reported studies, the less electronegative the halogen bond-donor the larger the stability of the halogen bond complex; and (ii) for any halide atom acting as halogen bond donor, the relative stability with respect to the R substituent groups follows the −X > −CF_3_ > −CHF_2_ > −CH_2_F > −CH_3_ trend, suggesting that the electron-withdrawing character of the R group affects the stability of the complexes. MP2 results were observed to correlate remarkably well with DFT values, indicating that the ωB97XD is capable of accurately describing the main interaction effects involved in the halogen bond formation of the studied systems. Analysis of the changes of NBO atomic charges indicated that an important bond polarization occurs in the R–X bond upon complex formation. This observation supports the well-established criterion on halogen bonding, which states that it is a non-covalent interaction driven mainly by electrostatic effects. The latter was confirmed by the graphical representation of the $\chi_{XC}^{\sigma }$ function computed with a 0.3 a.u. isovalue for the different R–Br⋯NH_3_ complexes, where it was observed that the lone-pair electrons of the halogen bond donor are localized in a toroid-like basin located at the R–Br sigma-axis. Moreover, the $\chi_{XC}^{\sigma }$ function plotted onto a 0.04 a.u. isovalue of the one-electron density showed that the $\chi_{XC}^{\sigma }$ value on the sigma-hole zone is small for all the studied systems, suggesting a high degree of electron delocalization (i.e., electron depletion). It was also observed that the $\chi_{XC}^{\sigma }$ value computed on the sigma-hole zone correlates with the electron-withdrawing character of the R-substituent group as well as the relative stability of the complexes. Finally, evaluation of the $\chi_{C}^{\sigma }$ function in [BrCN]_n_ chains with *n* = 2 to 6 showed that its value grows with the number of monomers considered in the system, which is an indication of a non-negligible contribution of dispersion effects in the stability of halogen bond systems.

## Figures and Tables

**Table 1:** Halogen bond distance (D**_X_**_⋯**N**_), change in the halogen bond acceptor, halogen bond donor, and its first neighbor NBO charges (Δq_N_, Δq_X_, and Δq_XN_), and binding energies computed at the DFT and MP2 levels of the different R–X⋯NH_3_ complexes. D**_X_**_⋯**N**_ and Δq values reported in parenthesis correspond to MP2 results. BE values in parenthesis correspond to the BSSE uncorrected energies. Binding energies obtained at the CCSD(T) /6-311+G(d,p)//MP2/6-31+G(d,p) are also reported. Distances, charges, and energies reported in Å, *e*, and kcal/mol, respectively.

Halogen Bond Donor	R Substituent	D_X__⋯N_	Δq_N_	Δq_X_	Δqx_N_	BE^DFT^	BE^MP2^	BE^CCSD(T)^
Cl	Cl	2.52 (2.58)	0.04 (0.00)	0.00 (0.05)	−0.10 (−0.10)	7.0 (7.9)	4.4 (6.0)	3.8
	CF_3_	2.97 (3.01)	−0.01 (−0.01)	0.03 (0.04)	−0.02 (−0.02)	2.8 (3.2)	2.5 (3.3)	2.5
	CHF_2_	3.05 (3.10)	−0.01 (−0.01)	0.03 (0.04)	−0.02 (−0.02)	1.7 (2.0)	1.5 (2.1)	1.5
	CH_2_F	3.24 (3.28)	0.00 (0.00)	0.01 (0.01)	−0.01 (−0.01)	1.0 (1.2)	0.7 1.3)	0.7
	CH_3_	3.27 (3.37)	0.00 (0.00)	0.02 (0.03)	−0.01 (−0.01)	−0.1 (0.0)	−0.4 (−0.1)	−0.3
Br	Br	2.55 (2.50)	0.04 (0.01)	0.02 (0.08)	−0.14 (−0.17)	8.7 (12.4)	7.0 (11.5)	5.0
	CF_3_	2.88 (2.91)	−0.01 (−0.02)	0.03 (0.05)	−0.03 (−0.03)	4.9 (7.1)	3.9 (6.5)	3.9
	CHF_2_	2.99 (3.03)	−0.01 (−0.01)	0.04 (0.05)	−0.03 (−0.03)	3.5 (5.2)	2.6 (4.7)	2.6
	CH_2_F	3.05 (3.11)	0.00 (−0.01)	0.03 (0.04)	−0.02 (−0.03)	2.4 (4.0)	1.6 (3.4)	1.6
	CH_3_	3.08 (3.17)	0.00 (0.00)	0.03 (0.04)	−0.02 (−0.03)	1.5 (3.0)	0.6 (2.2)	0.6
I	I	2.58 (2.62)	0.08 (0.02)	0.06(0.13)	−0.23 (−0.25)	16.4 (17.2)	10.6 (12.7)	8.7
	CF_3_	2.80 (2.84)	0.03 (−0.01)	0.01 (0.05)	−0.04 (−0.04)	11.8 (12.3)	9.5 (10.7)	8.9
	CHF_2_	2.94 (3.01)	0.03 (0.00)	0.03 (0.06)	−0.04 (−0.04)	7.7 (8.1)	5.7 (6.7)	5.4
	CH_2_F	3.07 (3.15)	0.02 (0.00)	0.03 (0.06)	−0.04 (−0.04)	5.1 (5.5)	3.4 (4.2)	3.2
	CH_3_	3.14 (3.26)	0.02 (0.00)	0.03 (0.05)	−0.04 (−0.04)	3.5 (3.9)	1.8 (2.5)	1.6

**Table 2:** MP2 binding energies, minimum value of the $\chi_{XC}^{\sigma }$ function mapped onto the one-electron density 0.04 a.u. isovalue surface and population of the Br valence lone-pair basin for the different R–Br⋯NH_3_ complexes considered in the present study. The electron population, Pop (Br), computed as the integral of the $\chi_{XC}^{\sigma }$ basin corresponding to the Br halogen bond donor lone pairs is also reported. BE^MP2^, $\chi_{XC}^{\sigma }$, and Pop (Br) values are reported in kcal/mol, bits and *e*, respectively.

Halogen Bond Donor	R Substituent	BE^MP2^	$\mathrm{Min}\;(\chi_{XC}^{\sigma })$	Pop (Br)
Br	Br	7.0	0.194	5.56
	CF_3_	3.9	0.259	5.64
	CHF_2_	2.6	0.269	5.69
	CH_2_F	1.6	0.275	5.73
	CH_3_	0.6	0.271	5.75
	CH_3_CH_2_	0.3	0.278	5.78
	CH_2_CH	1.2	0.270	5.77
	CHC	3.6	0.249	5.59

## Supplementary Materials

The following are available online. Figure S1: $\chi_{XC}^{\sigma }$ mapped onto the 0.04 a.u. isovalue of the electronic density for the R–Br⋯NH_3_ complexes (R = CF_3_, CHF_2_, CH_2_F, CH_3_, CH_3_CH_2_, CH_2_CH, and CHC). The range 0.2–0.5 goes from red to blue. For a sake of clarity, the NH_3_ molecule and map are hidden in the view of the lower part of the figures.
