# Supplementary material for: Theoretical Description of R–X⋯NH3 Halogen Bond Complexes: Effect of the R Group on the Complex Stability and Sigma-Hole Electron Depletion
Source: Molecules. 2020 Jan 25;25(3):530. doi: 10.3390/molecules25030530 (PMC7037998; doi:10.3390/molecules25030530)
Supplement: Supplementary file 1 [file molecules-25-00530-s001.pdf]

Supporting Information

The Information Content of the Conditional Pair Density as a Theoretical Tool for the Analysis of the Sigma Hole in Halogen Bonded Systems

Juan Zurita [1,2], Vladimir Rodriguez [1,2,3], Cesar Zambrano [1,2], Jose Ramón Mora [1,2], Luis Rincón [1,2] and F. Javier Torres [1,2,*]

Figure S1: χ_{XC}^{σ} mapped onto the 0.04 a.u. isovalue of the electronic density for the the R—Br ··NH$_3$ complexes (R = CF_3, CHF_2, CH_2F, CH_3, CH_3CH_2, CH_2CH, and CHC). The range 0.2 – 0.5 goes from red to blue. For a sake of clarity, the NH3 molecule and map are hidden in the view of the lower part of the figures.

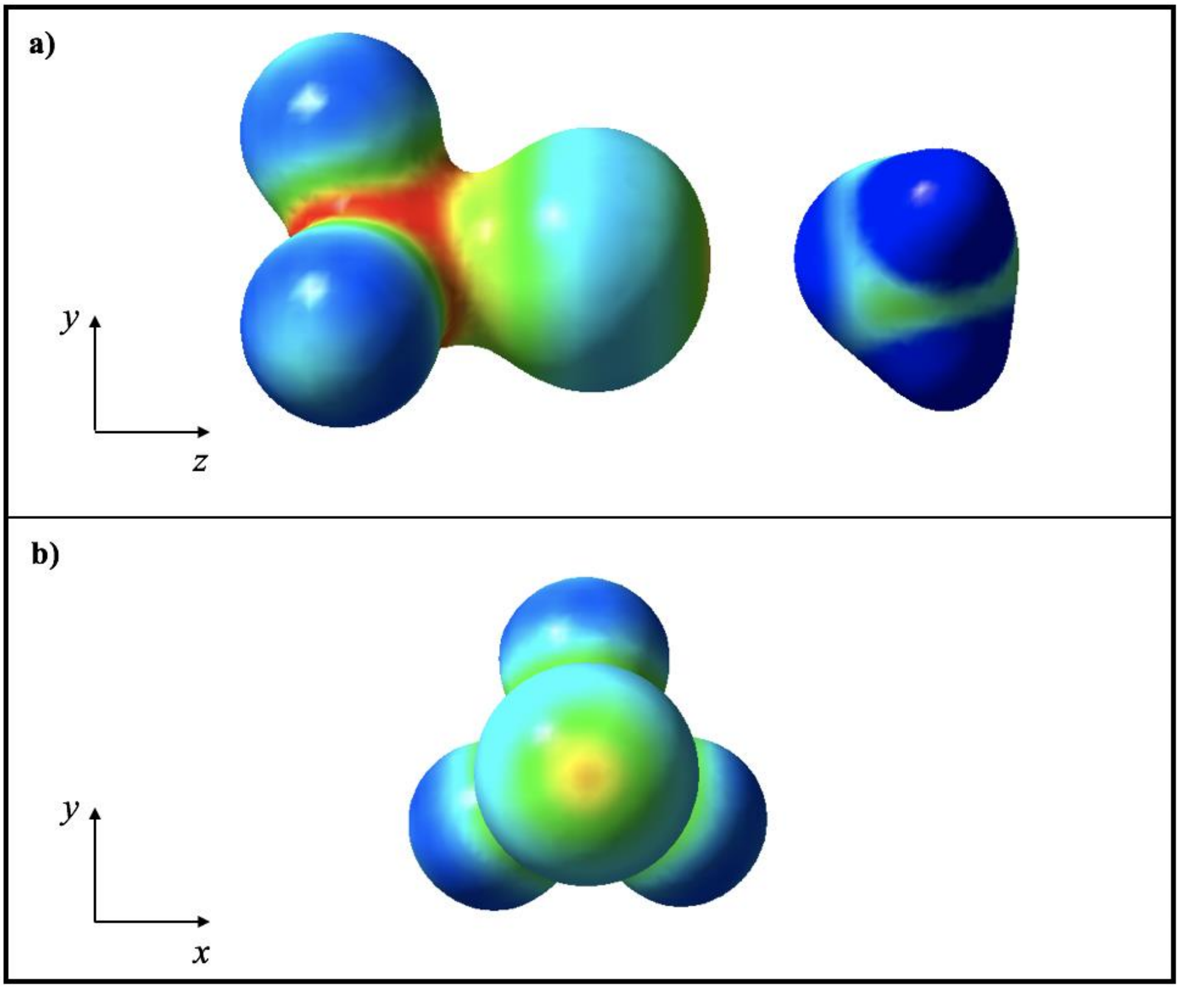

CF_3—Br ··NH$_3$

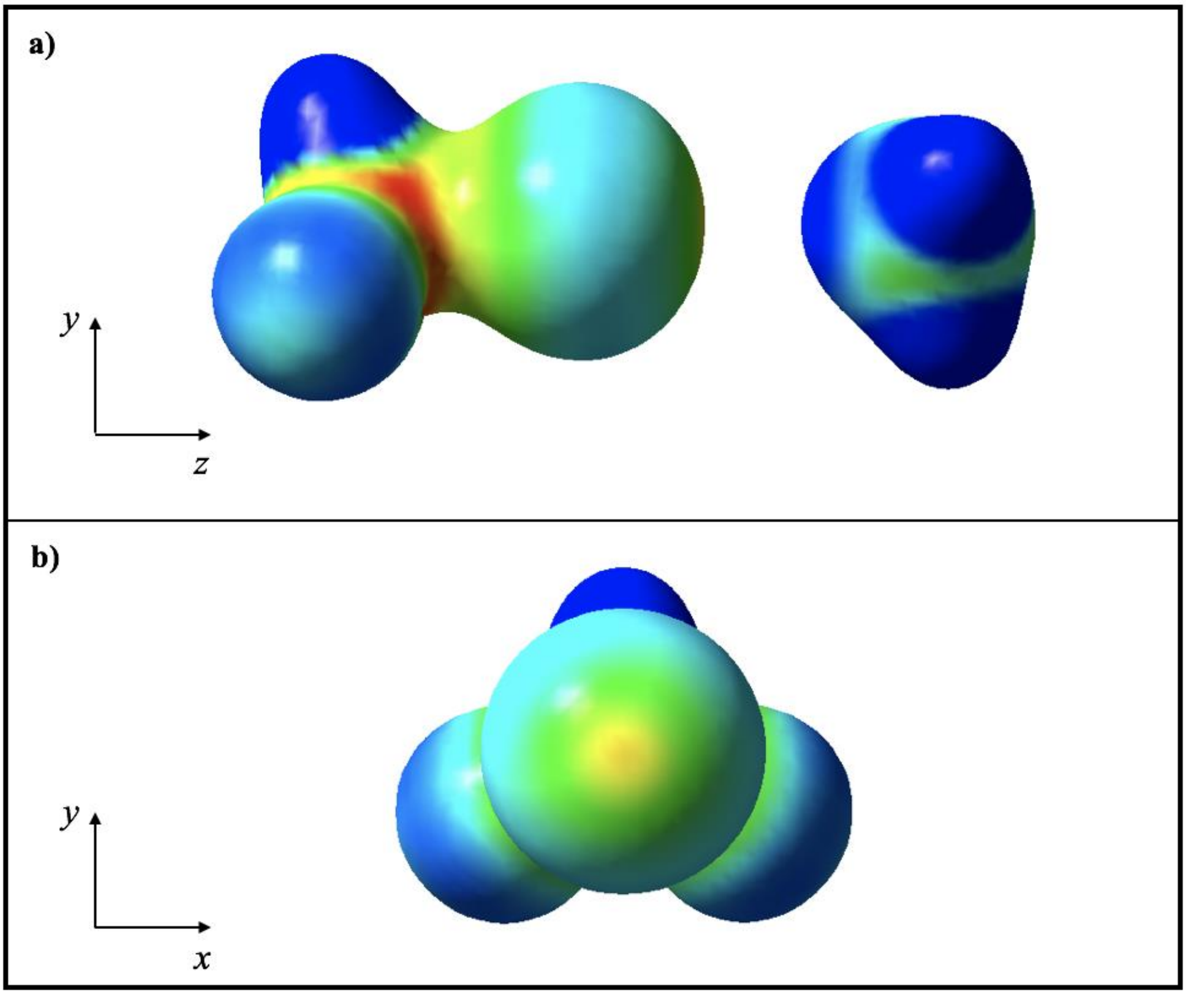

CHF_2—Br ···NH_3

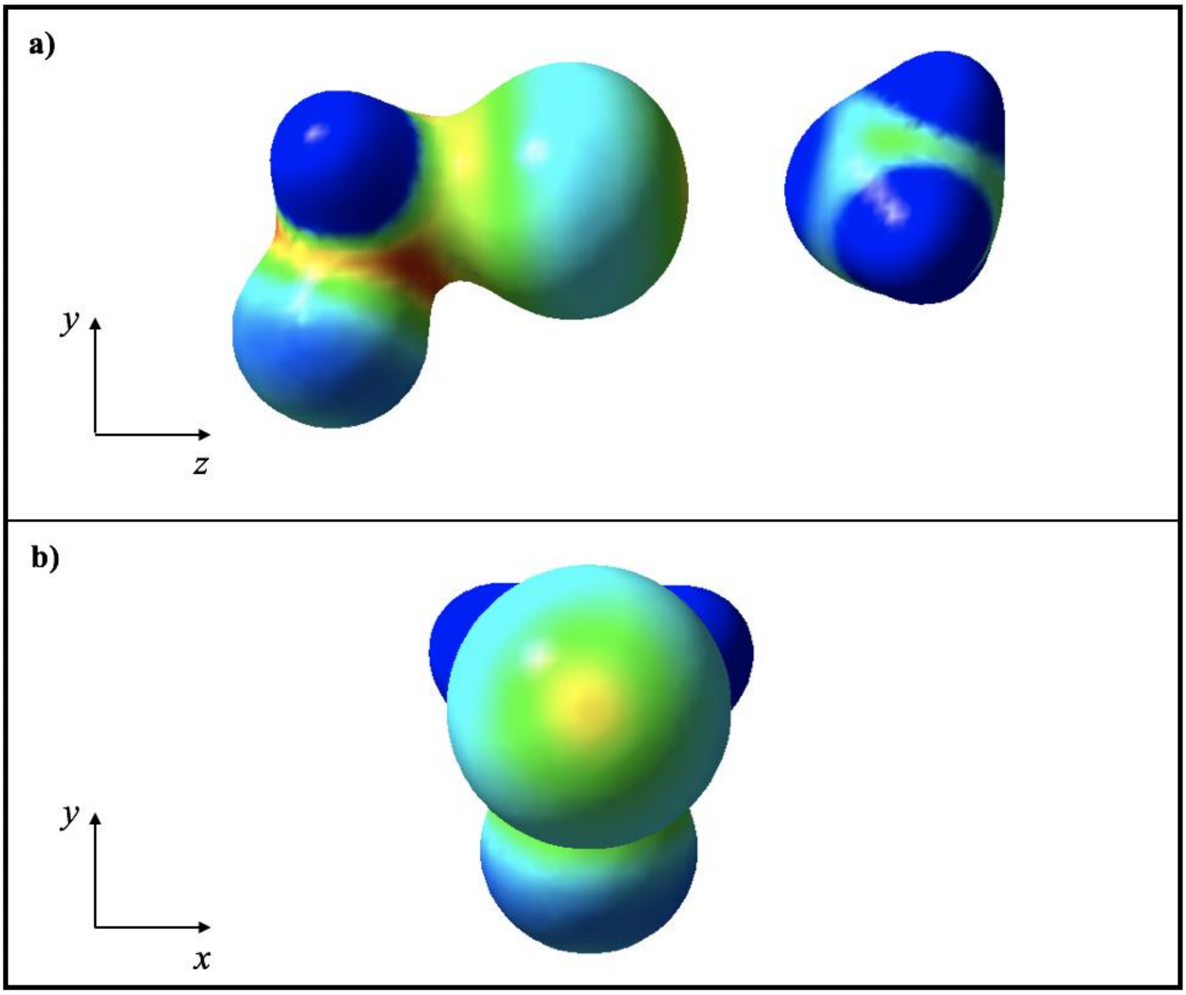

CH_2F—Br ···NH_3

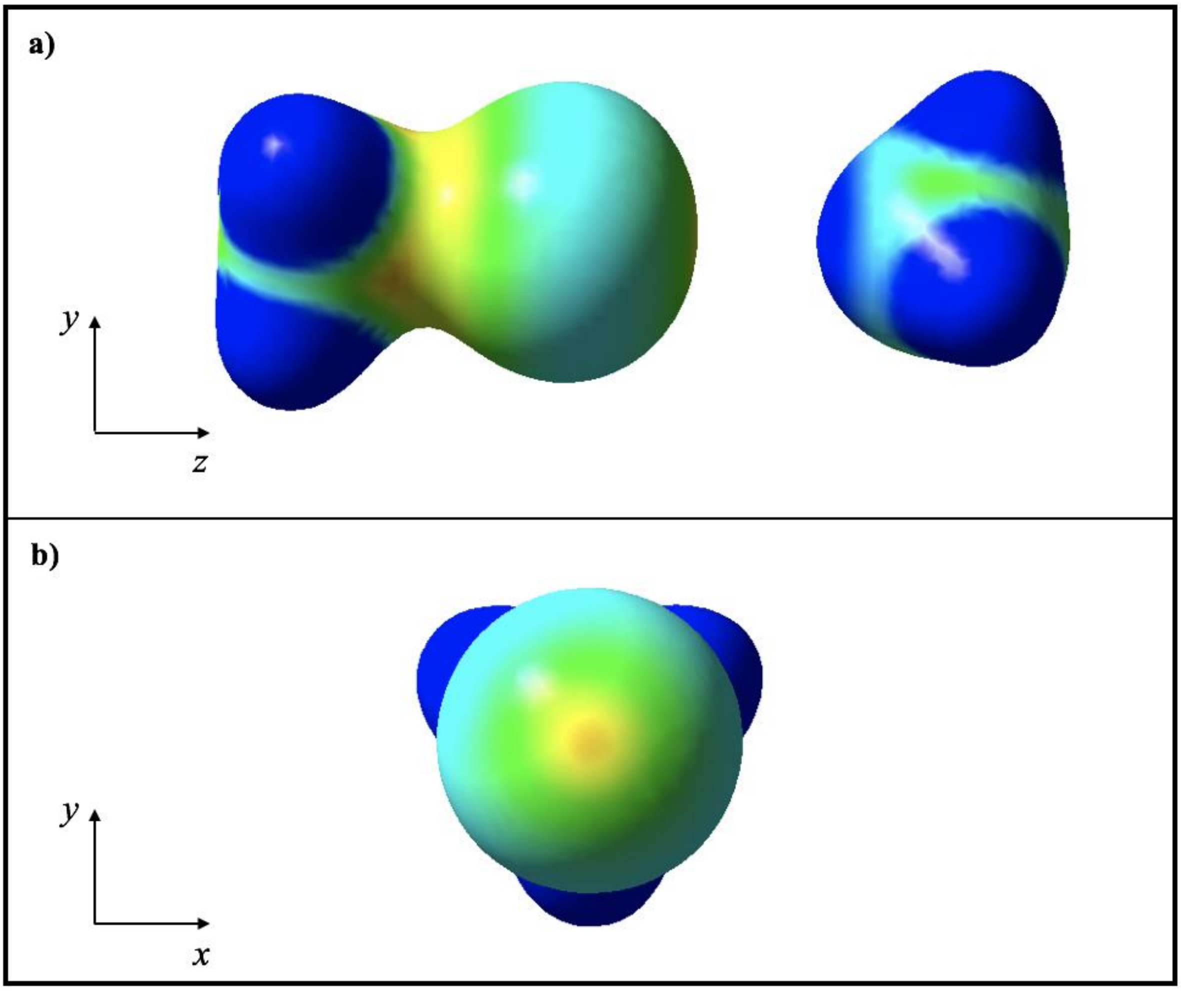

CH_3—Br ··· NH_3

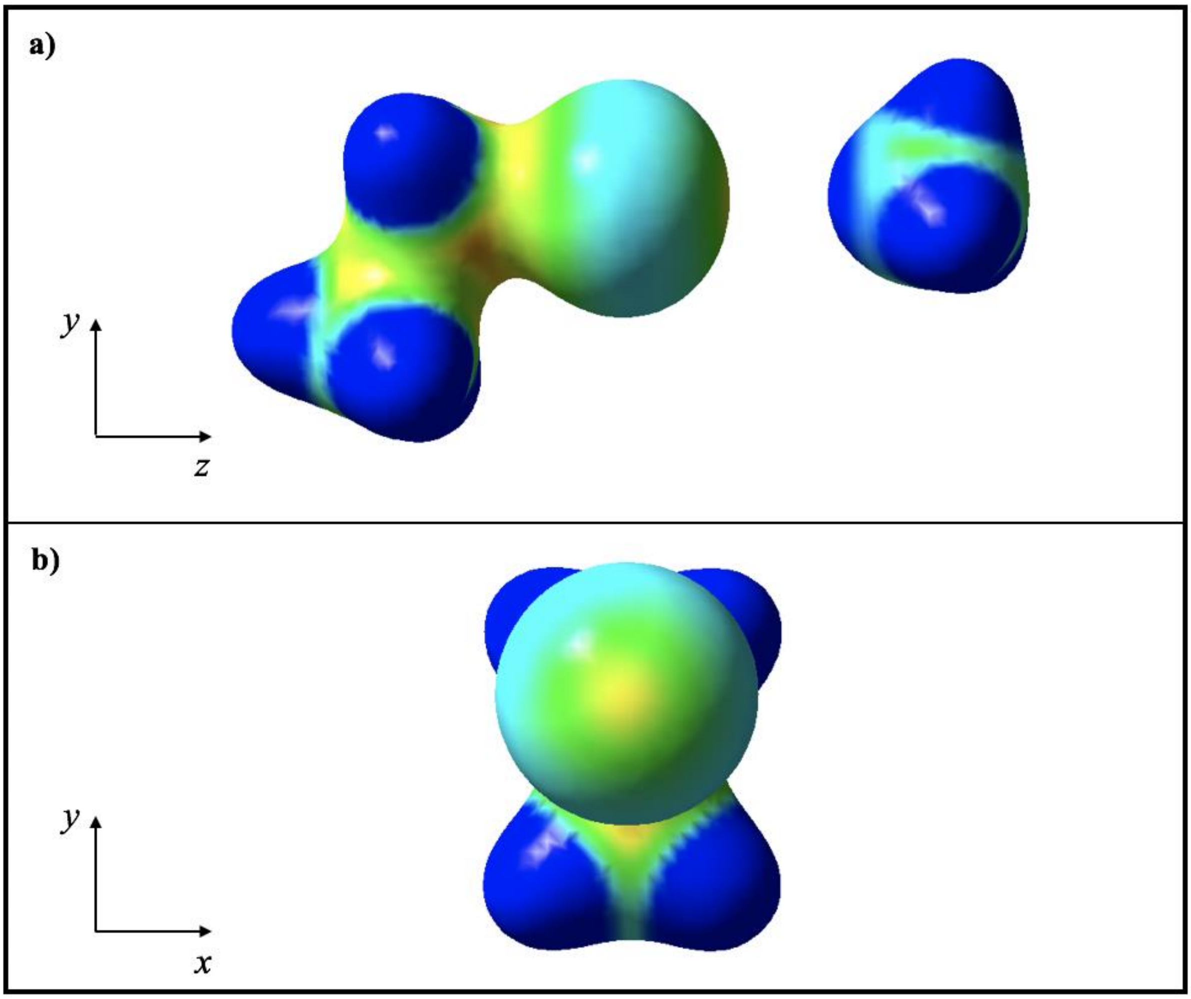

CH_3CH_2—Br ···NH_3

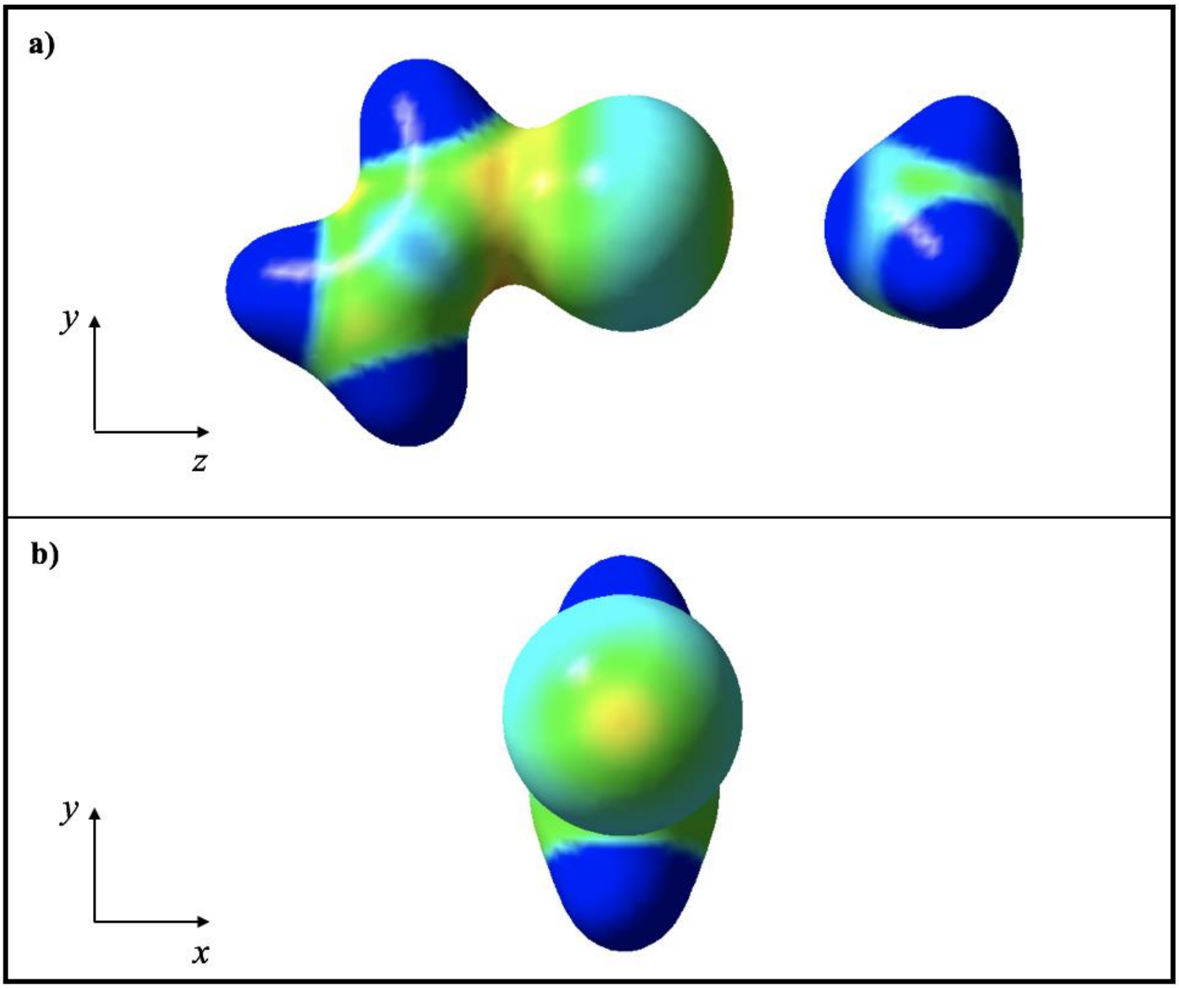

$CH_2CH—Br\cdots NH_3$

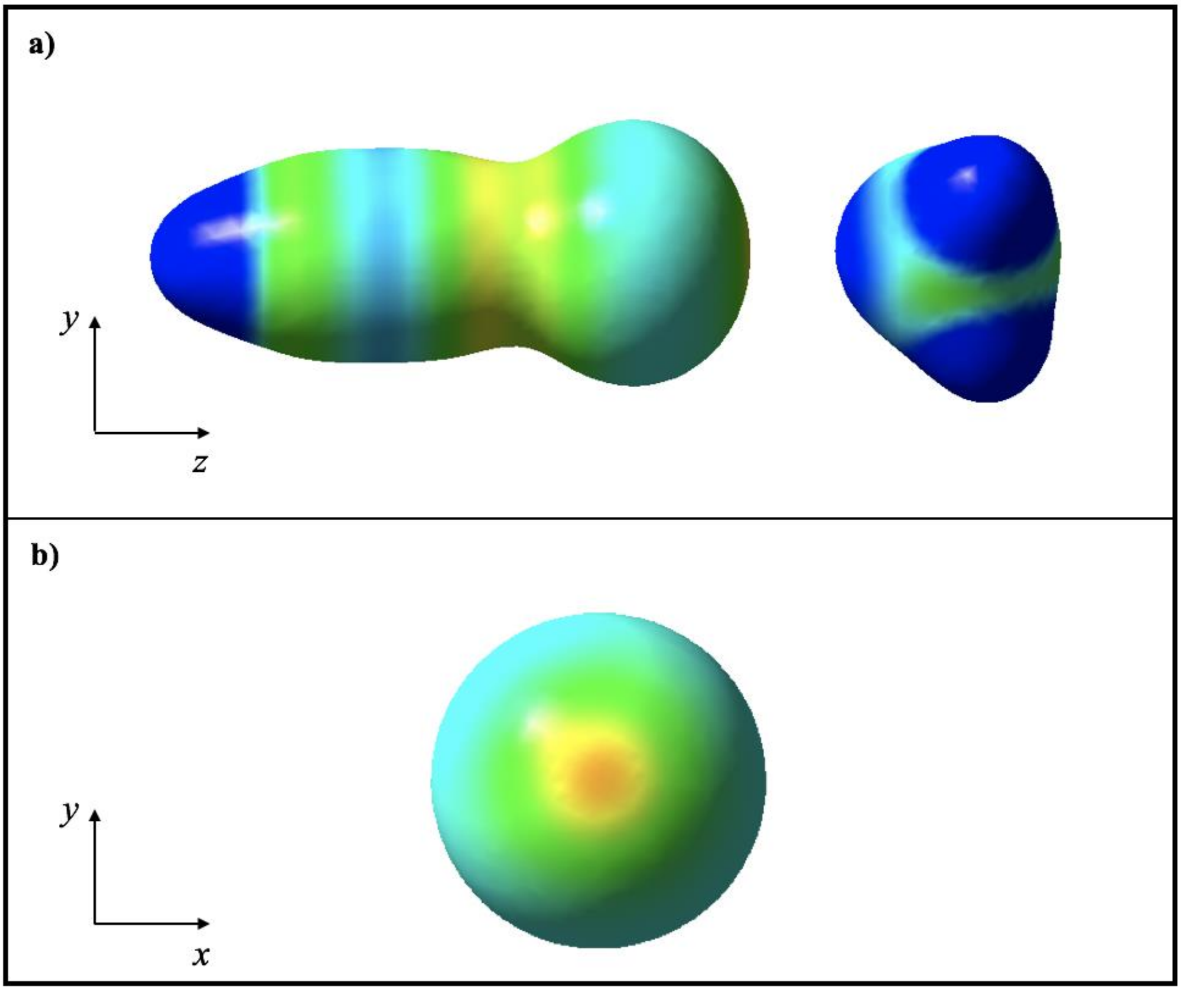

$CHC—Br\cdots NH_3$
